# #CPR challenge: Impact of a social media campaign on cardiopulmonary resuscitation awareness and skills among young adults − A quasi experimental study

**DOI:** 10.1016/j.resplu.2024.100711

**Published:** 2024-07-15

**Authors:** Prithvishree Ravindra, H.S. Shubha, Savan Kumar Nagesh, Rachana Bhat, Ankit Kumar Sahu, Sukriti Chugh, B.N. Lavanya, Padma Rani

**Affiliations:** a Center for CARE (Cardiac Arrest Research and Education), Department of Emergency Medicine, Kasturba Medical College, Manipal, Manipal Academy of Higher Education, Manipal, Karnataka 576104, India; b Manipal Institute of Communication, Manipal, Manipal Academy of Higher Education, Manipal, Karnataka 576104, India; c Department of Anesthesiology, Kasturba Medical College, Manipal, Manipal Academy of Higher Education, Manipal, Karnataka 576104, India; d Department of Emergency Medicine, All India Institute of Medical Sciences, New Delhi, Delhi 110029, India; e Kasturba Medical College,Manipal, Manipal Academy of Higher Education, Manipal, Karnataka 576104, India; f Manipal College of Health Professionals, Manipal, Manipal Academy of Higher Education, Manipal, Karnataka 576104, India

**Keywords:** CPR, Social media, Instagram Health education, Nomination based social media campaign, Bystander CPR

## Abstract

**Aim:**

The aim of our study was to explore the effect of nomination-based social-media campaign and CPR-skill-booth on change in knowledge as well as hands-only CPR skills among young adults.

**Methods:**

A quasi-experimental study was conducted in two non-healthcare-stream colleges, one intervention and other control arm. After baseline evaluation of CPR knowledge in both colleges, a 4-week nomination-based social media campaign ‘#CPR challenge’ was rolled out in the intervention arm which included a CPR-skill-booth that was setup for one hour every day to train interested participants in CPR. The participants were encouraged to share the same on their social media handles and data of self-reported metrics were collected. A post-intervention assessment was conducted in both arms, to assess knowledge and its translation to hands-only-CPR skills using qCPR mannequin and qCPR app® for objective assessment.

**Results:**

A total of 690 assessments were done; Intervention arm (pre-intervention-214, post intervention −155) and control arm (pre-intervention −157, post-intervention −134). The baseline knowledge scores were comparable in both groups. Knowledge score doubled in the intervention arm, (p < 0.001) from a median value of 29% (IQR:14 – 43) in the pre-intervention-cohort to 57% (IQR:29 – 71) in the post-intervention-cohort. Median CPR-skill-score was higher in the intervention arm 67.5(IQR:39–92) in comparison to control arm 21 (IQR:1–53) (p < 0.001). In terms of social media engagement, 50% of participants had watched the videos and 40.6% attended the CPR-skill-booth.

**Conclusion:**

Strategies such as a nomination-based social media campaign can improve the awareness, knowledge and also skills regarding hand-only CPR.

## Introduction

Despite significant strides in resuscitation science, the outcomes in Out-of-Hospital cardiac arrests (OHCA) remain dismal.1., 2. Immediate bystander Cardiopulmonary resuscitation (CPR) and early defibrillation remain the cornerstone for good outcomes in OHCA. Inadequate CPR literacy forms a major barrier to these crucial links in the chain of survival.3., 4., 5. Various community education initiatives have been rolled out to address this gap.

The advent of social media has revolutionized the dissemination of information. International scientific organizations have highlighted the potential role of social media in facilitating information exchange regarding cardiac arrest care and have also emphasized this to be a priority area for research.^6^ A good example of the social media reach is the “World Restart a Heart” initiative. In 2019, this global movement was able to train more than 5.4 million people, and the social media reach was up to 206 million people.^7^ Since then, this initiative has been garnering a positive response and deploying social media as one of the tools to create awareness about cardiac arrest.^8^

Although social media can reach a wider audience at a faster pace, the challenges in utilizing it for CPR education are ensuring the accuracy of shared information,^6^ engaging the content consumers, ensuring their learning, and understanding of CPR steps, and assessing the real-world impact.^6^ Moreover, the translation of visual learning of CPR through videos on social media, to actual performing of the skills needs to be studied.

'Social media challenges' are unique social media trends, that bank on exponential reach through a nomination process. One of the landmark public health-related 'social media challenge' was the “Ice Bucket Challenge”, which created a lot of buzz and raised awareness regarding Amyotrophic lateral sclerosis.^9^ The success of the Ice-bucket challenge raises the possibility of such challenges being useful in sparking interest in other health-related themes. Hence, we designed a nomination-based social media challenge titled #CPRChallenge and implemented it in a university college, along with a voluntary CPR skill-training booth. Our study explored its effectiveness in increasing knowledge, as well as hands-only CPR skills among the students.

## Methodology

The study was initiated after approval from the institutional ethics committee (IEC1: 311/2022) and Clinial Trial Registries India (CTRI) registration (2023/06/054547). The study was conducted in three phases: Phase 1: Baseline assessment; Phase II: Social media intervention; and Phase III: Post-intervention knowledge (IIIA) & skill assessment (IIIB). Two colleges from a non-healthcare stream, situated in the same district, were selected as the study centres. One college served as an intervention arm (Manipal Institute of Communication, Manipal) and the other college served as a control arm (Poorna Prajna College, Udupi). The intervention college had around 550 students, studying in the communication stream and control arm had around 500 students, studying Business Administration.

### Phase I: Baseline assessment

Baseline knowledge about CPR and confidence levels to perform CPR were assessed in both institutes using a digital questionnaire. The short questionnaire (Total of 15 questions) was reviewed by 5 experts (2 from Emergency Medicine, 2 from anaesthesiology, and 1 from critical care) and pilot-tested on 6 university students from a different non-healthcare stream (Supplementary annexure 1), however was not statistically validated. From the comprehensive list of college student registries, the college administration assigned students block-wise for data collection. The study investigators went to the classrooms and administered the digital questionnaire to all students who consented to be a part of the study. The questionnaire had an option for indicating prior formal training in basic life support/ CPR courses and those participants were excluded from analysis & phase III recruitment.

### Phase II: Social media intervention #CPR challenge

In the intervention arm college, a 4-week social media campaign was launched in August 2023. The students in college were encouraged to upload a video on social media demonstrating hands-only CPR and then nominate their classmates to do the same.

The recommended format for the social media #CPRChallenge was:

a. A personal anecdote/ bystander CPR statistics/ message with the importance of learning CPR
b. Steps to identify cardiac arrest
c. Hands-only CPR demonstration
d. Challenging other individuals from the institute to take the #CPRChallenge

As the most used social media platform by the students was Instagram, it was recommended to share their videos on this platform, and the same was used to collect the metrics.

To kickstart the campaign, the Head of the institute took the #CPRChallenge and challenged the students and faculty to take the challenge. The same was posted on the official Instagram page of the institute.

A social media booth was set up in a visible corner of the college. The booth was functional for one hour on working days (10 AM-11:00 AM), which was free time for the students. The booth consisted of two CPR mannequins, a table to perform CPR, a backdrop with the words #CPRChallenge #Learntosavelives, and two volunteers available to train the interested students in CPR and help them record their videos for social media. The students and faculty could visit the booth, of their own accord, to learn about CPR and shoot their video for their social media. The campaign was conducted for 4 weeks in the interventional arm college. No training or social media campaign was conducted in the control arm college during the above 4 weeks.

### Phase III: Post-intervention assessment

At the end of the campaign, a post-intervention assessment including knowledge assessment & skill assessment was conducted in the interventional arm to assess the effectiveness of the social media intervention. The same assessment was also conducted in the control arm, four weeks after the baseline assessment, to take into consideration, compare & study other potential factors that may have contributed to the improvement in knowledge, such as local factors/ news, baseline knowledge testing increasing the curiosity to learn about CPR or other unforeseen factors.

The assessment consisted of the same digital questionnaire used to assess the baseline knowledge levels along with a few additional questions regarding their participation in #CPRChallenge (the attendance at the booth, participating in the challenge, and viewing the CPR challenge on social media feeds). Participants for the post-intervention assessment were a fresh set of random students assigned from the comprehensive college student list (excluding students with prior training), who may or may not have participated in the baseline assessment. This technique of sampling was used to consider the cross-dissemination of knowledge via social media even in students who would not have participated in the baseline assessment. To assess the impact of social media knowledge translation into applicable skills, the participants were also asked to perform 1 min of hands-only CPR on the Laerdal q-CPR mannequin®, following the questionnaire. The quality of CPR was recorded on the Laerdal q-CPR app®. The average chest compression rate, mean chest compression depth, CPR score, percentage of time with correct chest compression rate, adequate chest compression depth, and good release were measured. CPR score is a composite score of Laerdal Medical which uses an algorithm considering the various parameters.^10^

The primary aim of the study was to compare the improvement in knowledge about CPR in the intervention in comparison with the control groups. Hence, the post-intervention sample size was calculated based on approximately 15% improvement in CPR knowledge among the intervention arm (25%) compared to control arm (10%), with an alpha error of 5% and power of 90%. The minimum sample size required was estimated to be 133 in each arm. Accounting for the potential dropouts during post-intervention skill assessment, a sample size of 160 students was selected from the student list of each arm using an online computer software. Among these, students who had undergone other structured CPR training courses in the previous 2 years (3 in the intervention group, none in control group) and students not consenting to take part in the study were excluded.

The secondary objective was to compare the CPR skills among the two groups, using the data obtained from the Laerdal q-CPR mannequin®. Additionally, data was collected regarding the number of participants visiting the booth, learning CPR and recording the video, and self-reported social media metrics of their posts at the end of 1 week (views, likes, comments, shares, as applicable) were taken from the participants who shot the video. This was done to assess the reach of the social media campaign.

All the information was collected through the online questionnaire and spreadsheet. Quantitative variables were tested for normality using Kolmogorov-Smirnov test. Normally distributed quantitative variables were depicted as mean and standard deviation, and significance of difference was analysed using paired and unpaired Student t-tests. Non-normally distributed quantitative variables were expressed as median and interquartile range (IQR), and these were analysed using Wilcoxon signed Rank test (paired samples) and Mann Whitney *U* test (between intervention and control arm). Categorical variables were expressed in terms of frequency and percentage and analysed using Chi-square test. All statistical tests were two-tailed and a p-value of less than 0.05 was considered significant. All the analysis and graphs were prepared in IBM SPSS for Windows (Version 26, Armonk, NY: IBM Corp)**.**

## Results

**Fig. 1:**
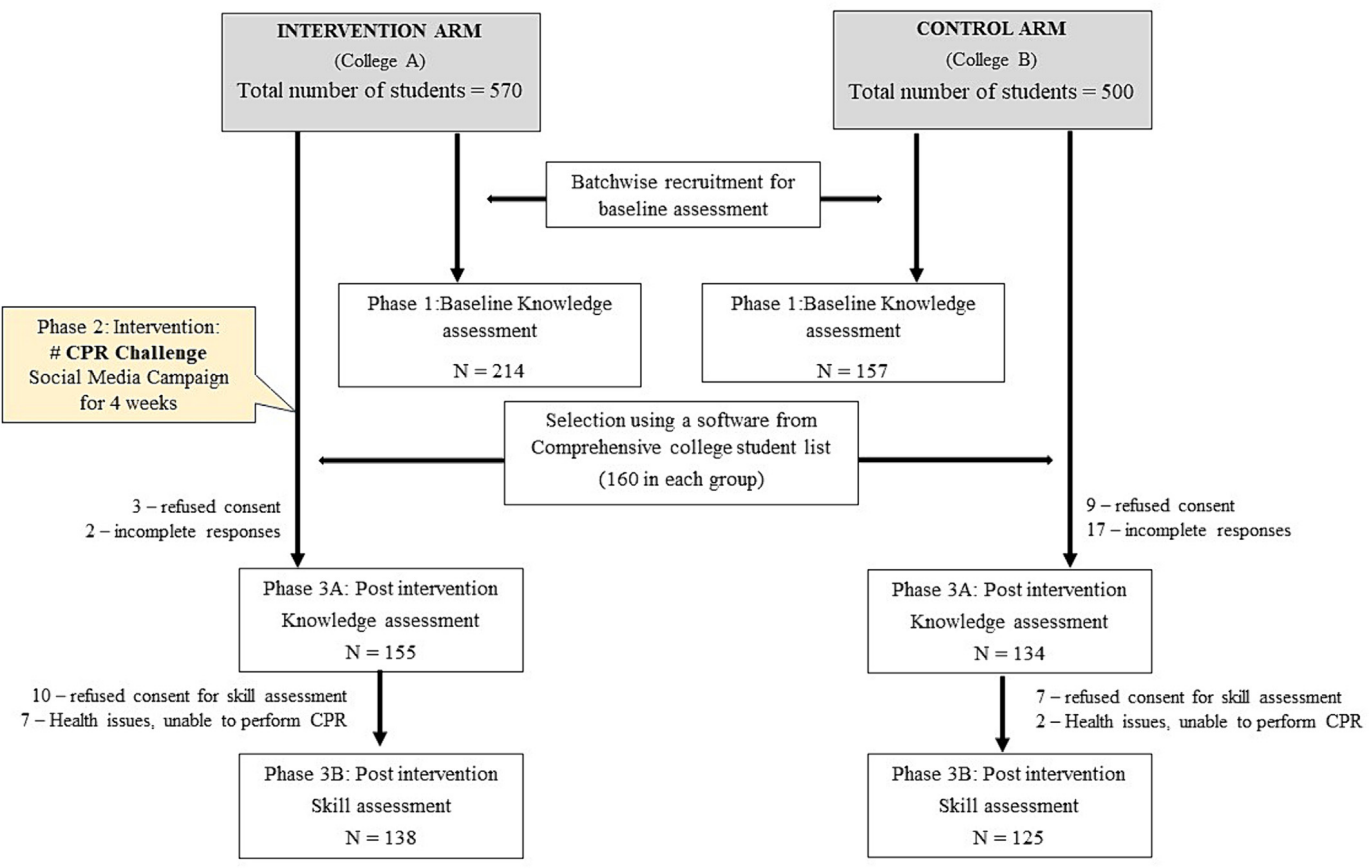
CONSORT Flowchart of study recruitment.

In Phase I of this study, 214 students were assessed in the intervention arm and 157 students were assessed in the control arm. In phase III of the study, a fresh random set of 160 students were included in the post-intervention assessment. Following refusals and exclusion of incomplete responses, 155 student questionnaires were analysed in the intervention arm and 134 students were analysed in the control arm. Further 17 students in the intervention arm and 9 in the control arm dropped out from the skills analysis (Fig. 1: CONSORT diagram). There was some overlap of students who took part in both Phase I and Phase III of the study.

### Phase I: Baseline data

The study population consisted of college students within the age group of 18–22 years. There was no significant difference in the baseline knowledge and demographics in both groups (Supplementary Table 1).

### Phase III A: Post-intervention knowledge assessment

The social media campaign #CPR challenge was effective in improving knowledge about CPR. Students in the intervention group had a median score of 57% in the Knowledge score (% of questions answered correctly) as compared to the 29% in the control group. There was a clear increase in the knowledge score in the intervention arm following the intervention when compared to the baseline (57% vs 29%). This change was not noted in the control arm (29% vs 29%) demonstrating a positive effect of the social media campaign.

### Phase III B: Post-intervention skill assessment

**Fig. 2:**
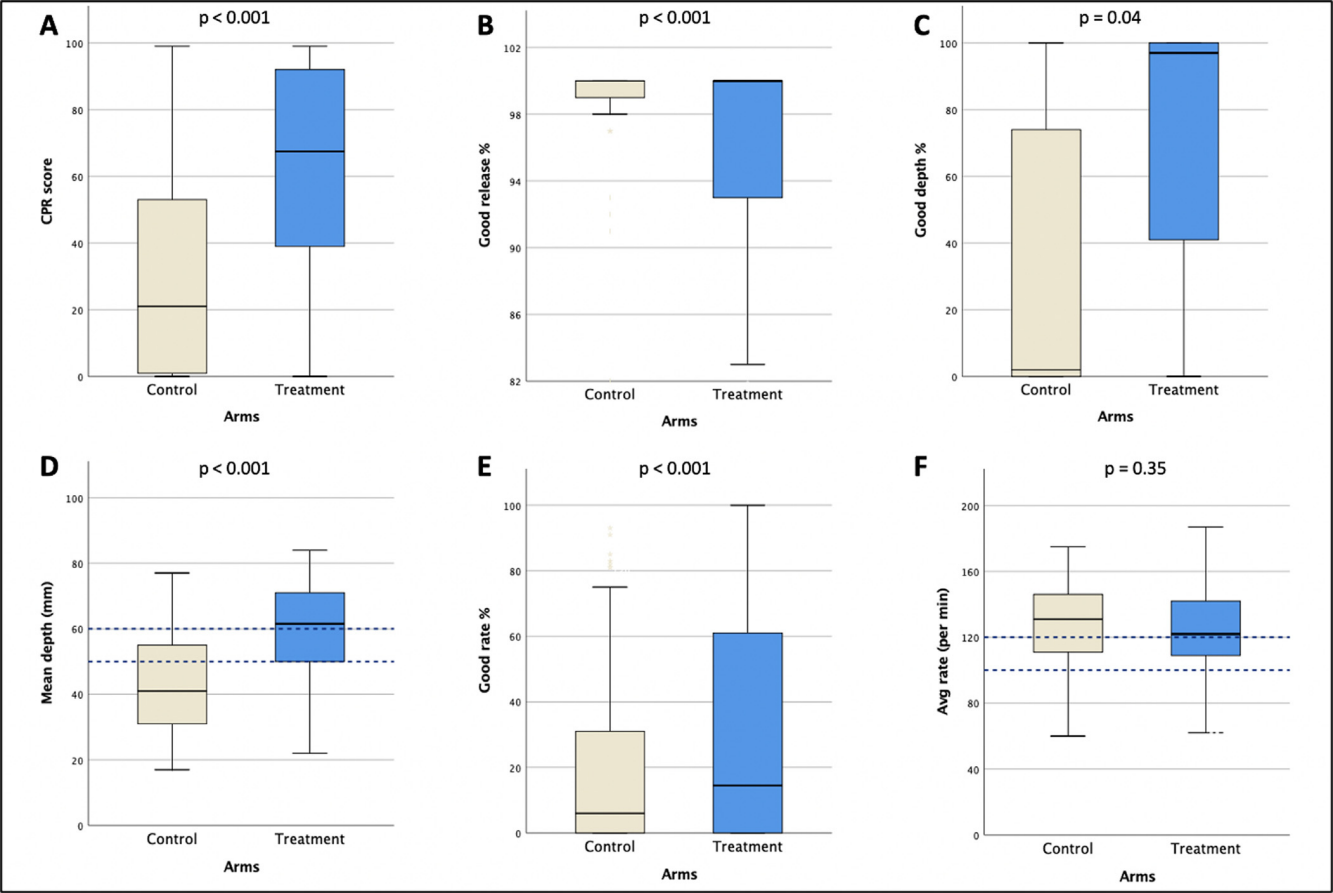
Figure 2 shows comparison of chest compression matrices among the treatment versus control arm. The CPR score, good release %, good depth %, mean depth and good rate % were significantly higher in the intervention arm as compared to the control arm.

The effect of the intervention on the CPR skills was studied. The skill analysis was done in both the intervention (n = 138) and control arm (n = 125). The proportion of students with correct hand placement during CPR was significantly higher (p < 0.001) in the intervention arm (81%), compared to the control arm (34%). Similarly, median CPR score, good depth %, mean depth and good rate % were higher in the intervention arm as shown in Fig. 2 and Supplementary Table 2. Overall, it was found that apart from the 'good release' matrix, all other CPR matrices had satisfactory scores. The average rate of chest compression was similar in both arms.

**Fig. 3:**
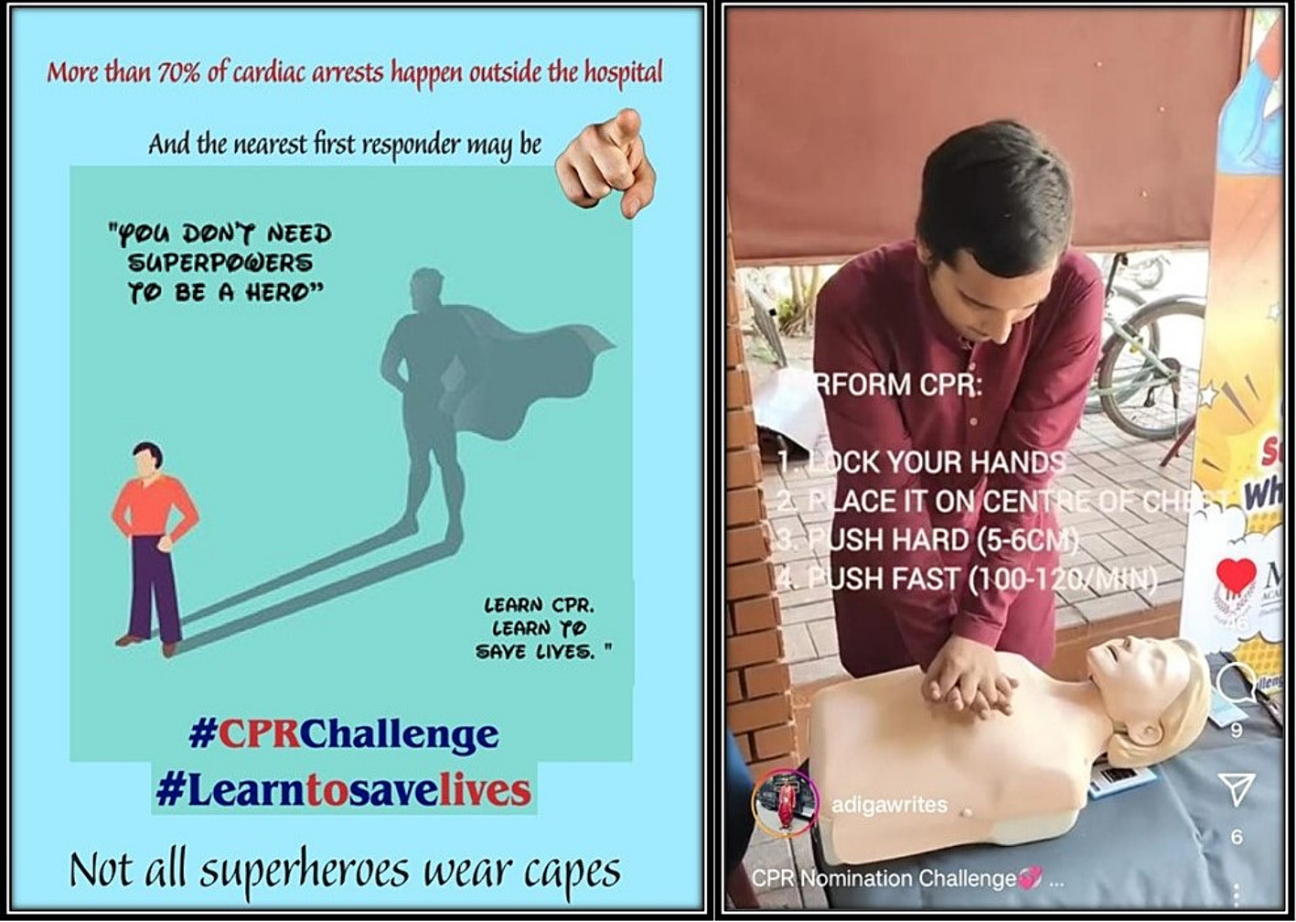
CPR-skill-booth set up at the Intervention college.

Similarly, the attitude of the students (i.e., the willingness to provide CPR in cardiac arrest and willingness to attend CPR training) improved significantly as shown in Table 1. On a scale of 1–5, students expressed an increase in confidence in identifying a cardiac arrest and performing CPR in a time of need (p < 0.001).

**Table 1:** Demographic and Pre-Intervention and Post-Intervention assessment parameters in both study groups.

	Intervention arm (n = 369)			Control arm (n = 291)		
**Characteristics**	**Pre-intervention** **(n − 214)**	**Post-intervention** **(n − 155)**	**p −value**	**Baseline** **(n = 157)**	**Post-4 week** **(n = 134)**	**p −value**
Age (years)	19 (18–20)	20 (18–21)	0.39	20 (18–21)	21 (18–22)	0.23
Gender:						
Female	154 (72)	107 (69)	0.41	91 (58)	69 (51.5)	0.27
Male	60 (28)	48 (31)		66 (42)	65 (48.5)	
Heard about term CPR	214 (100)	154 (99.4)	0.24	127 (80.9)	103 (76.9)	0.4
Aware when a person requires CPR	157 (73.4)	135 (87.1)	< 0.001	82 (52.2)	68 (50.7)	0.8
Knowledge score %	29 (14–43)	57 (29–71)	< 0.001	29 (14–43)	29 (14–30)	0.3
Willing to do CPR	145 (67.8)	113 (72.9)	0.03	86 (54.8)	77 (57.5)	0.86
Will to attend training	193 (90.2)	127 (81.9)	0.02	131 (83.4)	112 (83.6)	0.41
Confident in identifying arrest (scale of 1–5)	2 (1–3)	3 (2–3)	< 0.001	2 (1–3)	2 (1–3)	0.68
Confident in performing CPR (scale of 1–5)	2 (1–2)	3 (2–3)	< 0.001	1 (1–3)	2 (1–3)	0.31

### Reach of the social media campaign

During the four-week social media campaign in the intervention group, a total of 104 CPR skill booth participations were recorded (Fig. 3). Social media engagement indices were measured at the end of 4-week campaign, which suggested 27 Instagram uploads (4 Instagram reels & 23 stories). There were further engagements with an average of 1423 views, 114 likes, 12 shares & 5 comments per upload. The stories had an average of 362 views. The maximum engagement was observed for the Instagram reel shared by the head of the institute which garnered 4113 views and 22 shares.

During the post-intervention knowledge and skill assessment, it was noted that 40.6% (56 out of 138 in the intervention arm) had attended the CPR skill booth in their institute, and 50% students had watched the videos posted on social media. 29% students indicated they had not been exposed to either. Whereas, in the control arm, where no active campaign was conducted, 28.8% (36 of 125 in control arm) of students revealed their exposure to the concept of CPR through various sources which included online sources, television, movies and personal experiences.

The confounding impact of having a physical CPR training booth cannot be overlooked even though the booth was active for a very short duration during the day (1 h). Hence, we investigated the differential effect of attending skill booth and watching CPR video posted on social media. It was found that the chest compression matrices (correct hand position, median CPR score, good depth % and mean depth) were significantly higher among the students who attended CPR booth as well as watched the videos, as compared to those who had utilized only one modality or no modalities of training (Supplementary Table 3).

The perceived barriers to providing CPR in a public setting were surveyed in all responses (n – 660). A total of 455 (69%) responses suggested that they would not be able to provide CPR because of no prior training, and 304 (46%) responses indicated perceived harm to the patient with CPR. Gender barriers, spread of communicable diseases, harm to self and cultural barriers were other responses noted as barriers, as shown in Table 2.

**Table 2:** Perceived barriers to initiate CPR.

**Perceived barriers to initiate CPR #**	**Total (N = 660)** **n (%)**	**Intervention arm (N = 369); n (%)**	**Control arm** **(N = 291); n (%)**
No prior training	455 (68.9)	287(77.8%)	168 (57.8%)
May harm the patient	304 (46.1)	210 (57%)	94 (32.3%)
Gender barrier	119 (18)	84 (22.8%)	35 (12%)
Spread of infections such as COVID	54 (8.2)	38 (10.2%)	16 (5.5%)
May harm the self	45 (6.8)	24 (6.5%)	21 (7.2%)
CPR will not be useful	11 (1.7)	7 (1.9%)	4 (1.4%)
Religious or cultural barriers	10 (1.5)	5 (1.4%)	5 (1.7%)

The data is representative of all responses including baseline (Phase I) & Phase IIIA (post-intervention) questionnaire response. Total responses analysed were 660; 369 (214 + 157) in Intervention arm and 291 (157 + 134) in control arm.

# − Multiple responses were allowed in the questionnaire.

## Discussion

This trial demonstrates the positive effect of an educational social media campaign on the knowledge, and cardiopulmonary resuscitation skills among a group of non-healthcare stream college students when compared to a similar demographic that did not participate in the social media campaign.

Social media can be a potential tool for widespread dissemination of essential information. Its strengths as an educational tool are its millions of diverse users that improve its reach, the integration of multiple media formats of texts, pictures, and videos, and its ability to use social influence to inspire behavioural change.

We used targeted strategy to maximise benefit from using the social media platform. Firstly, we conducted the study among college-going students aged 18–24, who are the most active demographic on social media sites.11., 12. Secondly, we encouraged students to upload videos sharing personal stories about cardiac arrest, steps to identify cardiac arrest, and performing hands-only CPR. We anticipated higher engagement with such content and hoped to improve knowledge retention as such videos were a form of peer-to-peer information exchange.^13^ Thirdly, we used the hashtag #CPR challenge where an individual could nominate their friends to share their video demonstrating hands-only CPR adding a component of gamification. Lastly, we also requested university heads and teachers to participate in the #CPR challenge as we thought they would be important “opinion leaders”. The social media metrics in our study were limited compared to campaigns such as ‘World Restart a Heart’ initiative; however, we estimated metrics only over a week compared to over years such as in the above initiatives.^8^

The American Heart Association in its scientific statement in 2016 has acknowledged the potential applications of social media in improving emergency cardiovascular and cerebrovascular care such as public education and engagement. Campaigns such as “The Heart Truth”, “Call Fast, Call 911″ and ”#WeAreHeart“ have successfully spread the knowledge about cardiac arrest using social media.^6^ Similarly, a quasi-experimental study conducted by Ziabari et al demonstrated the positive role of using modern communication networks in improving knowledge levels regarding basic life support. In this study, participants who underwent CPR training received continuous distance education via Telegram for three months. This led to a better knowledge score as compared to a control group that didn't receive such social media updates.^14^ Our study also demonstrated a doubling of knowledge scores after the social media challenge (29% to 57%).

While the effect of education via social media on knowledge has been studied previously, research is scant on whether such modalities improve the psychomotor skills of CPR among recipients and translate to increased initiation of bystander CPR in a cardiac arrest scenario.^15^ Previous studies have shown that, video-based training for chest compression-only life support resulted in a notable 35% reduction in the response to compression time, when contrasted with traditional instructor-led training, without any change in other aspects of effective CPR.^16^ However, the distinction between video-based self-directed CPR training and social media CPR would lie in the authenticity of shared content and the clarity of explanations; The delivery of content is standardized in the former in contrast to varied authenticity and explanation styles with the use of social media, where laypersons may contribute to content. Our study tried to address this important question by assessing the quality of hands-only CPR following our social media educational campaign. We found that the interventional arm performed better than the control arm with respect to correct hand position, good rate percentage, good depth percentage and overall CPR score. We can infer from this finding that there is a gap between understanding “how to perform” hands-only CPR and developing the necessary skills to perform good quality CPR. This is further highlighted by the finding that participants who attended the CPR booth where they received hands-only CPR training on a mannequin, had better chest compression scores than those who only watched the video. Other CPR learning kiosks like the ones described by Moskalyk et al and Chang PM et al have shown clear benefits in the form of performing high-quality CPR (19% had a CPR score of 70% or higher) and deeper and longer-lasting understanding of CPR (30% of participants).17., 18. Another benefit of the kiosk we set up was that videos uploaded from the kiosk would be screened for accuracy of shared information by the CPR-trained volunteers who manned the kiosk and helped record the video.

The success of such a campaign is encouraging to help similar campaigns on a larger scale. A combination of nomination-based social media such as the #CPR challenge with CPR learning kiosks at multiple sites can help rapidly raise awareness and CPR literacy at a local and global level.

The limitations which we perceive in our study were the limited time duration of social media campaign i.e., only for 4 weeks due to resource limitations. Such social media campaigns usually start slow and need time, rising awareness to become viral. The study population is a homogenous population targeting a single social media platform (Instagram). The use of a CPR skill booth to assist might have been a confounder, overestimating the impact of social media campaign. Generalisation of learning about CPR through social media across varied age groups, social media platforms and social backgrounds cannot be made from our findings and need larger studies to assess the feasibility and impact of such campaigns. Also, the results may not extrapolate into whether the increased knowledge and skills will translate into an increase in the percentage of bystander CPR given or an overall decrease in mortality in OHCA.

## Conclusion

Strategies such as a nomination-based social media campaign like the #CPR challenge can improve awareness and knowledge regarding hand-only CPR. There can also be a small improvement in CPR skills when combined with CPR learning kiosks. Such a multidimensional social media campaign can help raise awareness and CPR literacy rapidly.

## CRediT authorship contribution statement

**Prithvishree Ravindra:** Writing – original draft, Supervision, Methodology, Data curation, Conceptualization. **H.S. Shubha:** Writing – review & editing, Supervision, Project administration, Conceptualization. **Savan Kumar Nagesh:** Writing – original draft, Visualization, Validation. **Rachana Bhat:** Writing – review & editing, Writing – original draft, Visualization, Methodology, Data curation, Conceptualization. **Ankit Kumar Sahu:** Writing – original draft, Validation, Methodology, Formal analysis. **Sukriti Chugh:** Resources, Methodology, Data curation. **B.N. Lavanya:** Resources, Supervision, Data curation. **Padma Rani:** Supervision, Resources.

## Declaration of competing interest

The authors declare that they have no known competing financial interests or personal relationships that could have appeared to influence the work reported in this paper.
