# Supplementary material for: #CPR challenge: Impact of a social media campaign on cardiopulmonary resuscitation awareness and skills among young adults − A quasi experimental study
Source: Resusc Plus. 2024 Jul 15;19:100711. doi: 10.1016/j.resplu.2024.100711 (PMC11304058; doi:10.1016/j.resplu.2024.100711)
Supplement: Supplementary Data 1 [file mmc1.docx]

Supplementary Table 1: Median Pre & post intervention scores in the study groups

|  | **Overall** | **Intervention arm** | **Control arm** | **p - value** |
| --- | --- | --- | --- | --- |
| Pre-intervention score | 2 (1 - 3) | 2 (1 - 3) | 2 (1 - 3) | 0.12 |
| Pre- intervention score % | 29 (14 - 43) | 29 (14 - 43) | 29 (14 - 43) | 0.12 |
| Post- intervention score | 2 (1 - 4) | 4 (2 - 5) | 2 (1 - 2) | < 0.001 |
| Post- intervention score % | 29 (14 - 57) | 57 (29 - 71) | 29 (14 - 30) | < 0.001 |

Supplementary Table 2: Post-intervention skill assessment scores in the study groups

|  | **Overall (n - 263)** | **Control (n - 125)** | **Intervention (n - 138)** | **p - value** |
| --- | --- | --- | --- | --- |
| **Correct hand position** | 115 (43.7) | 34 (27.2) | 81 (58.7) | < 0.001 |
| **CPR score** | 47 (12.5 - 81) | 21 (1 - 53) | 67.5 (39 - 92) | < 0.001 |
| **Good release %** | 100 (98 - 100) | 100 (99 - 100) | 100 (93 - 100) | 0.21 |
| **Good depth %** | 62 (0 - 99) | 2 (0 - 74) | 97 (41 - 55) | < 0.001 |
| **Mean depth (mm)** | 52 (38.5 - 66.5) | 41 (31 - 55) | 62 (50 - 71) | < 0.001 |
| **Good rate %** | 8 (0 - 46.5) | 6 (0 - 31) | 15 (0 - 61) | 0.04 |
| **Avg rate/ min** | 126 (110 - 143) | 131 (111 - 146) | 122 (109 - 142) | 0.09 |

Supplementary Table 3: Comparison of skill assessment scores with social media engagement in the Intervention study group

|  | **Attended skill booth and watched video (n - 27)** | **Attended skill booth only**  **(n - 29)** | **Watched video on social media (n - 40)** | **None**  **(n - 42)** | **p - value** |
| --- | --- | --- | --- | --- | --- |
| **Correct hand position** | 23 (85.2) | 23 (79.3) | 24 (60) | 11 (26.2) | < 0.001 |
| **CPR score** | 74 (40 - 96) | 90 (64 - 94) | 77 (40 - 93) | 47 (26 - 68) | 0.001 |
| **Good release %** | 100 (95 - 100) | 99 (93 - 100) | 100 (97 - 100) | 100 (70 - 100) | 0.78 |
| **Good depth %** | 99 (82 - 100) | 98 (88 - 100) | 96 (14 - 100) | 71 (11 - 99) | 0.02 |
| **Mean depth (mm)** | 64 (58 - 76) | 65 (56 - 70) | 57 (49 - 72) | 54 (42 - 65) | 0.04 |
| **Good rate %** | 17 (0 - 83) | 13 (2 - 61) | 16 (0 - 60) | 5 (0 - 44) | 0.39 |
| **Avg rate/ min** | 115 (106 - 142) | 118 (108 - 136) | 122 (109 - 137) | 130 (113 - 148) | 0.24 |

**Annexure 1: CPR Knowledge questionnaire for assessment**

**Knowledge- based** *(Questions 2-9 used for calculating knowledge score for comparison)*

1. Have you heard of Cardiopulmonary resuscitation/ CPR?

a. Yes

b. No

2. Are you aware about when does a patient require to be given cardiopulmonary

resuscitation?

a. Yes

b. No

3. In case of an emergency, which number will you call for help in your city?

a. 911

b. 108

c. No specific number

d. Don’t know

4. If you find a person who is not responding, what will be your next step after calling for help?

a. Start CPR

b. Check for pulse and breathing

c. Put the person on left side

d. Give key/ metal to their hand

e. Don’t know

5. Where will you check pulse in an adult

a. In the side of windpipe in neck

b. Wrist

c. Groin

d. Elbow

e. Don’t know

6. How long will you check pulse and breathing before starting CPR in an unresponsive patient?

a. less than 5s

b. 5-10s

c. 10-20s

d. 1 min

e. Don’t know

7. Where will you place the heel of your hand while performing CPR?

a. Lower half of Left side of chest

b. Upper half of breastbone (center of chest)

c. Lower half of breastbone (center of chest)

d. Upper half of left side of chest

e. Don’t know

8. What is the rate of chest compressions?

a. 60-100/min

b. 100-120/min

c. 80-100/min

d. 40-60/min

e. Don’t know

9. At what depth should chest compressions occur?

a. 2cm- 3cm

b. 3cm – 4cm

c. 4cm – 5cm

d. 5cm – 6 cm

e. Don’t know

**Attitudes and Perceptions**

10. Which of the following do you perceive as barriers to initiate CPR (tick as many as you agree with)

a. No prior training

b. May harm patient

c. May harm self

d. Gender barrier (unwilling to perform CPR on opposite gender)

e. Spread of infection such as COVID

f. Will not be useful

g. Religious/ cultural barriers

h. None of the above

i. Others:

11. On a scale of 1-5, how confident are you to be able to identify cardiac arrest (1- not at all

confident, 5 – very confident)

12. On a scale of 1-5, how confident are you to be able to perform hands-only cardiopulmonary

resuscitation (1- not at all confident, 5 – very confident)

13. Are you willing to perform CPR if you come across a patient in cardiac arrest? Yes/No/ Don’t know

14. Are you willing to attend a training program to learn and get certified in Basic life support

and CPR in the next 6 months (yes/no/don’t know)

15. Have you undergone structured training in CPR previously?

a. No

b. Yes, in the last 2 yr

c. Yes, between last 2-5 yr

d. Yes, more than 5 yrs ago

**Post #CPRchallenge additional questions**

16. Did you see the videos of CPR demonstration on social media? Yes/No

17. Did you see the demo in the challenge booth? Yes/ No

18. Did you upload video of #CPRchallenge on social media? Yes/ No
